# Fiber optic Fabry–Perot sensor that can amplify ultrasonic wave for an enhanced partial discharge detection

**DOI:** 10.1038/s41598-021-88144-4

**Published:** 2021-04-21

**Authors:** Haoyong Li, Jian Bu, Wenli Li, Jiaming Lv, Xiejun Wang, Kejia Hu, Yiting Yu

**Affiliations:** 1 Key Laboratory of Micro/Nano Systems for Aerospace (Ministry of Education), Northwestern Polytechnical University, Xi’an, 710072 China; 2 Key Laboratory of Micro- and Nano-Electro-Mechanical Systems of Shaanxi Province, Northwestern Polytechnical University, Xi’an, 710072 China; 3 Ningbo Research Institute, Northwestern Polytechnical University, Ningbo, 315040 China

**Keywords:** Optical sensors, Techniques and instrumentation

## Abstract

Ultrasonic wave is a powerful tool for many applications, such as structural health monitoring, medical diagnosis and partial discharges (PDs) detection. The fiber optic extrinsic Fabry–Perot interferometric (EFPI) sensor has become an ideal candidate for detecting weak ultrasonic signals due to its inherent advantages, and each time with a performance enhancement, it can bring great application potential in broadened fields. Herein, an EFPI ultrasonic sensor for PDs detection is proposed. The sensing diaphragm uses a 5-μm-thickness and beam-supported structure to improve the responsive sensitivity of the sensor at the resonant frequency. Furthermore, the ability of the sensor to detect characteristic ultrasonic signal of PDs is further enhanced by assembling a Fresnel-zone-plate (FZP)-based ultrasonic lens with the sensing probe to amplify the ultrasonic wave before it excites the sensing diaphragm. The final testing results show that the originally developed sensor owns the sensitivity of − 19.8 dB re. 1 V/Pa at resonant frequency. While, when the FZP is assembled with the probe, the sensitivity reaches to − 12.4 dB re. 1 V/Pa, and leads to a narrower frequency band, which indicates that the proposed method has a great potential to enhance the detection ability of sensor to characteristic ultrasonic wave of PDs.

## Introduction

Ultrasonic wave plays an important role in various scenarios ranging from industry to disaster monitoring benefiting from its good directionality, safety, and strong penetration. Detecting ultrasonic wave in power systems to find initial partial discharges (PDs) is one of its typical applications in the field of disaster monitoring, and one obvious advantage is that the position of a PD can been located via studying the amplitude attenuation or the phase delay of the received ultrasonic waves^1–4^. PDs detection based on ultrasonic waves have been widely studied. Piezoelectric transducers (PZTs) have been the preferred method for detecting PDs in power systems, due to the advantages of ease to install and replace. However, PZTs can be affected by electromagnetic interference (EMI). In the contrast, fiber optic extrinsic Fabry–Perot interferometric (EFPI) sensors are the ideal candidate for detecting weak ultrasonic signal in harsh environments because of many outstanding advantages, such as immunity to EMI, remote signal transmission, compact size, light weight, and easy installation^5–7^. The early EFPI sensors for PDs detection were proposed by Deng^8^.

On this basis, EFPI acoustic sensor has attracted considerable attention, and to improve its performance, a variety of EFPI sensors with different sensing diaphragms have been reported. Up to date, the sensing diaphragms based on silicon^5^, silver^6^, silicon dioxide^9^, polymer^10^, graphene^11^, gold^12^ and other materials have been successfully manufactured and demonstrated. Moreover, a wide range of structures, such as circular^9–12^, beam-supported^13^, micro-cantilever beam^14^ and silver three leaf clover diaphragm^15^ have also been designed and validated. In addition, Jo et al*.* developed an EFPI acoustic sensor using a photonic-crystal silicon diaphragm, which can improve the performance of sensor by enhancing the interference quality of Fabry–Perot cavity^16^. Overall, most of the reported studies focused on how to improve the performance of sensing diaphragms. Due to the wave nature of light and sound, acoustics can be amplified and focused like the light. In general, acoustic lenses are based on refraction, which are constructed from a homogeneous material with a curved surface, but the refractive lenses are usually bulky and high-cost because of requiring highly precise surface as well as the limited acoustic materials. Fresnel zone plates (FZPs) are an easy-to-manufacture and low-cost acoustic focusing device, which uses the alternative rigid and open apertures to construct interference of diffractive waves^17–19^. As a basic means of wave manipulations, acoustic focusing, including ultrasonic focusing, has been widely applied in various fields, such as non-destructive evaluation, underwater seabed mapping and biomedical imaging. However, to the best of our knowledge, the application of an acoustic lens to acoustic sensors for improving their performance has not been reported.

In this paper, a novel ultrasonic sensing system based on a microelectromechanical systems (MEMS) extrinsic Fabry–Perot interferometric (EFPI) is proposed, where the deformable mirror is made of a metallized 5-μm-thick silicon diaphragm, and the second mirror is the end surface of a fiber optic patch cord (PC). The silicon diaphragm uses a beam-supported structure, and is manufactured by utilizing the MEMS processing technology on a silicon-on-insulator (SOI) wafer. Furthermore, a method of using the FZP ultrasonic lens to improve the performance of the sensor is proposed and experimentally verified. The final testing results show that when the FZP ultrasonic lens is assembled with the sensing probe, the sensitivity increases by 7.4 dB and reaches to − 12.4 dB re. 1 V/Pa, and it indicates that the suggested method can bring a great application potential for PDs detection.

## Design and fabrication

### Design and fabrication of sensing diaphragm

**Figure 1:**
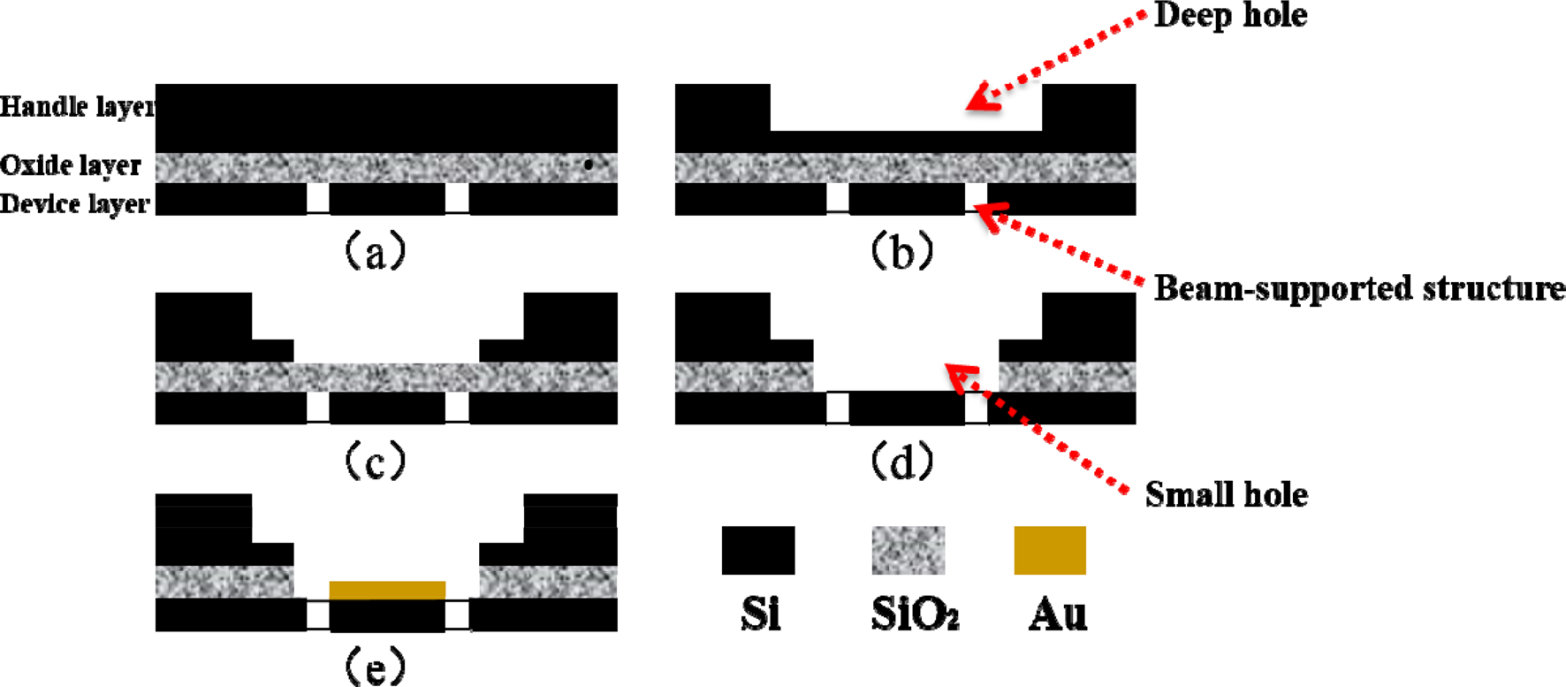
The schematic of manufacturing process for the sensing diaphragm: (**a**) etching beam-supported structure in the device layer of a SOI wafer, (**b**) etching a deep hole in the handling layer, (**c**) etching a small hole in the handling layer, (**d**) removing the oxide layer to free the sensing diaphragm, (**e**) sputtering a gold film on the sensing diaphragm as a reflective mirror.

**Figure 2:**
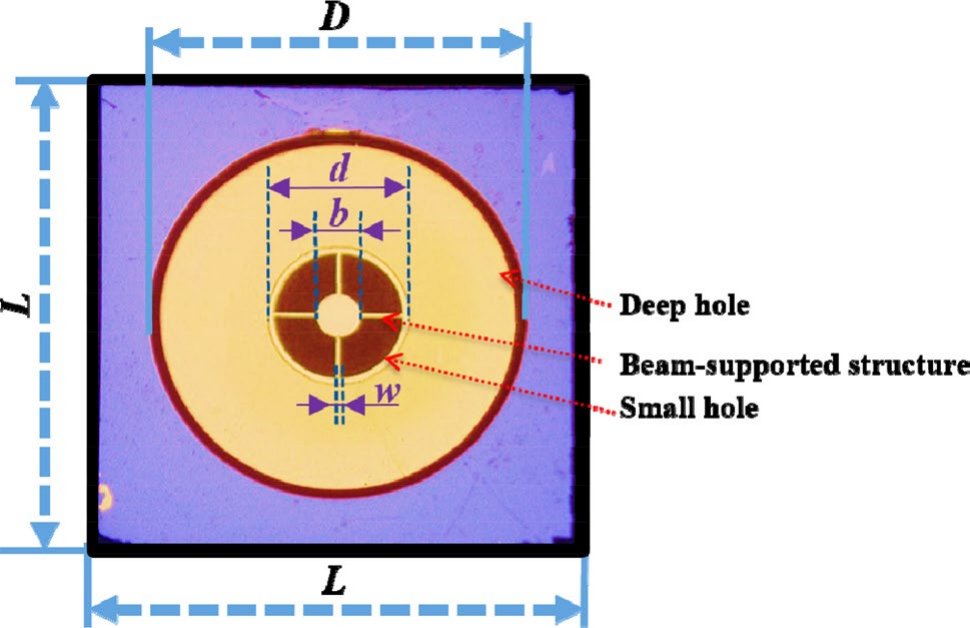
The microscopic photo of the beam-supported diaphragm.

**Figure 3:**
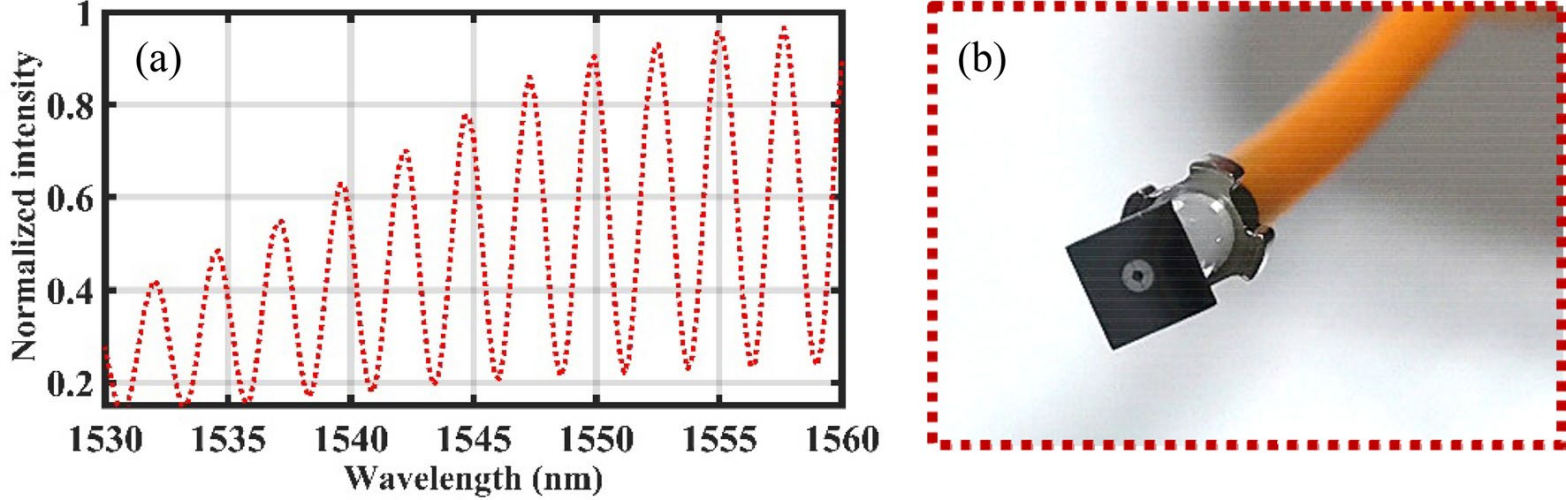
(**a**) The interference spectrum of Fabry–Perot (FP) cavity. (**b**) The finally assembled sensing element.

In our previous research, we proposed a beam-supported structure for fiber optic Fabry–Perot sensor, which has been proven to have great potential for ultrasonic detection due to low damping ratio^13^. In this paper, a beam-supported diaphragm with the natural frequency of 40 kHz is designed by using the finite element simulation software (ANSYS, ver. 14.5), and is manufactured on a SOI wafer (handling layer 500 μm, oxide layer 1 μm and device layer 5 μm) by employing the MEMS processing technology. The detailed fabrication process of the proposed diaphragm is illustrated in Fig. 1, which consists of five process steps: (1) Etching beam-supported structure in the device layer by utilizing the deep reactive ion etching (DRIE) process (Fig. 1a). (2) Etching a deep hole for the fiber sleeve with the depth of 120 μm in the handling layer also via the DRIE process, and its diameter is 2500 μm (Fig. 1b). (3) Etching a small hole with the diameter of 840 μm as the Fabry–Perot cavity in the handling layer again via the DRIE process, which is coaxial with the deep hole and stopped at the SiO_2_ layer (Fig. 1c). (4) Completely etching the SiO_2_ layer above the beam-supported diaphragm via the buffered oxide etching (BOE) process (Fig. 1d). (5) Sputtering a gold film (thickness: 100 nm) onto the beam-supported diaphragm to form a reflective mirror of the Fabry–Perot cavity (Fig. 1e). Figure 2 shows the microscopic photo of the final beam-supported diaphragm, and its sizes are given in Table 1. Finally, a fiber optic PC (type: straight tip, corning: SMF-28e, outside diameter: 2500 μm) is inserted into the deep hole to achieve accurate assembling of the Fabry–Perot cavity by using an alignment apparatus (a 25 nm step in vertical direction and 10 μm in horizontal two directions). The final reflection spectrum of the FP cavity is shown in Fig. 3a, and the assembled sensing element is shown in Fig. 3b.

**Table 1:** Geometrical dimensions of beam-supported diaphragm.

*L* (μm)	*D* (μm)	*d* (μm)	*b* (μm)	*w* (μm)
3500	2500	840	280	20

### Design and fabrication of FZP

**Figure 4:**
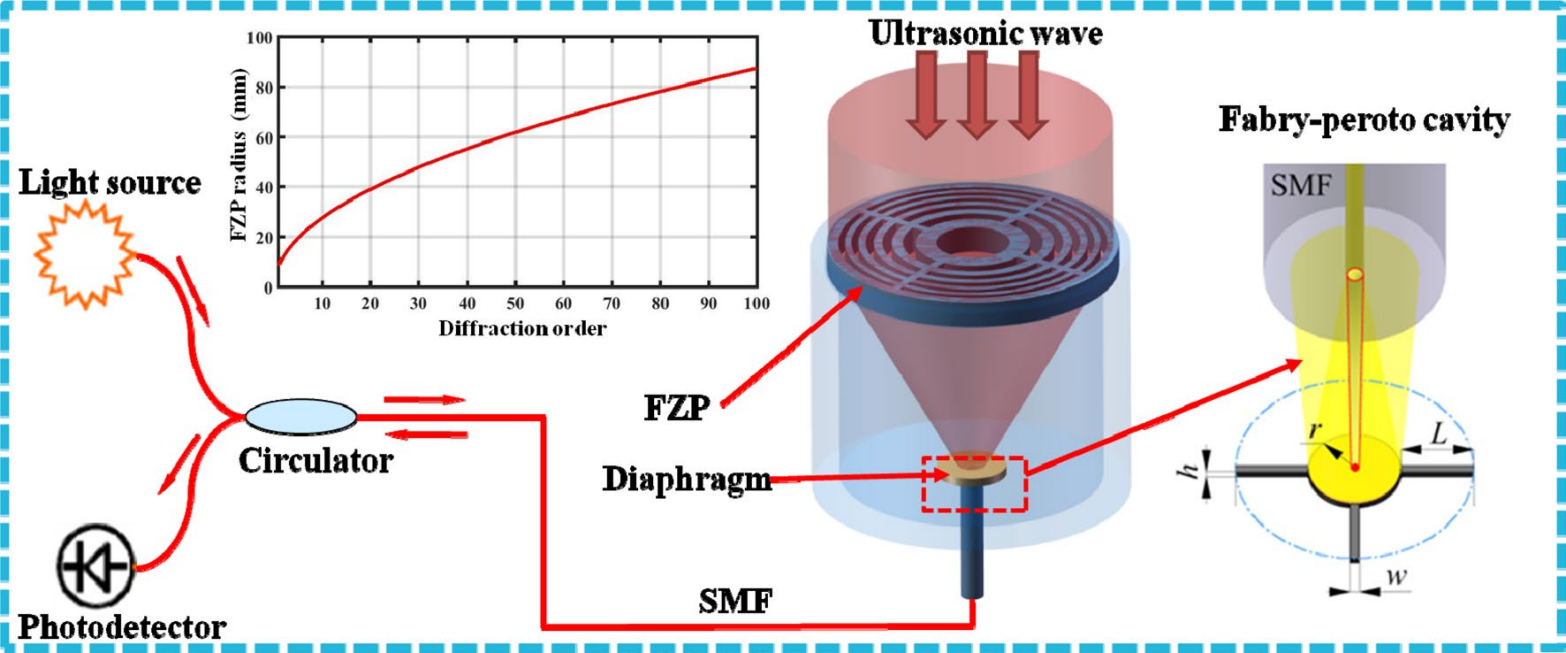
The schematic of the developed ultrasonic sensing system, with the inset showing the FZP radius as the diffraction order increasing and the Fabry–Perot cavity.

**Figure 5:**
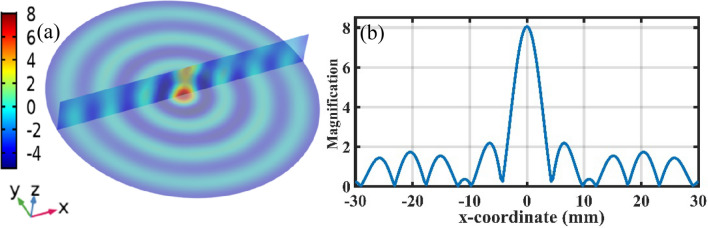
(**a**) Ultrasonic pressure distribution of the FZP in the focal and the z-axis plane. (**b**) The magnification of the FZP along the x-axis of the focal plane.

**Figure 6:**
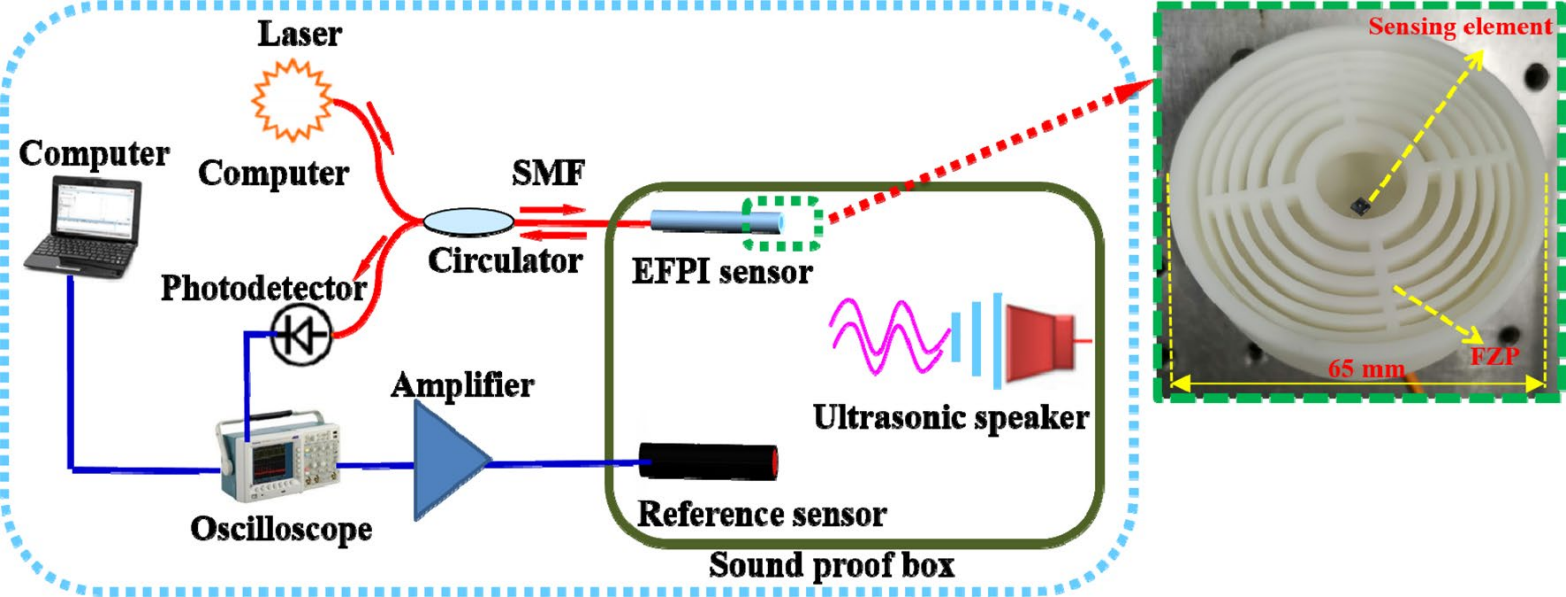
The experimental setup to characterize the proposed ultrasonic sensor, with the inset showing the image of the combined structure of sensing element and FZP.

FZP is a circular diffraction grating, with radially varying line period such that the diffracted ultrasonic waves forms a focal spot at some distance *F* from the FZP, and its ring radius can be calculated by:

1 $$ r_{i} = \sqrt {i\lambda F} $$

where *λ* is the wavelength of ultrasonic wave, and *i* is the diffraction order (1,2,3…). In general, the more diffraction orders are, the larger magnification of the lens will be. But the size of the final system also increases with the increasing diffraction orders, as shown in Fig. 4. In this paper, considering the size of the final sensing probe, we designed an ultrasonic lens with twelve diffraction orders (*i* = 12). According to the frequency characteristics of ultrasonic waves released by PDs, the focusing frequency of FZP lens is designed to be 40 kHz. The sensing principle of the proposed sensor was based on the well-known fiber optic Fabry–Perot interferometer, and the sensing diaphragm is placed at the focal spot of the FZP to pick up the ultrasonic waves as shown in Fig. 4. Further, we use the finite element method to calculate the performance of the proposed ultrasonic lens installed in the sensing probe. Figure 5a shows the ultrasonic pressure distribution of the FZP in the focal and the z-axis plane, and it can magnify to 8 times for 40 kHz ultrasonic wave at the focal spot as shown in Fig. 5b, which gives the magnification of the FZP on the x-axis of the focal plane. Finally, the proposed FZP is manufactured by using the 3D printing technology, and the inset of Fig. 6 shows the final combined structure of sensing element and FZP.

## Experimental validation

The performance of the developed sensor was characterized by comparison with a reference sensor, and the schematic diagram of the experimental setup is shown in Fig. 6, which is mainly consisted of an EFPI sensor, a 1550 nm narrow linewidth laser (stability: < 1% (1 h)), a fiber optic circulator (FOC), a photoelectric detector, a reference microphone and its amplifying circuitry (BSWA, MK401-MV401), an oscilloscope (Tektronix, MDO3024), a soundproof box, an ultrasonic loudspeaker (Core morrow) and a computer. Noting that to achieve the same ultrasonic pressure, the EFPI sensor and the reference sensor are fixed at two symmetrical positions relative to the ultrasonic loudspeaker in the soundproof box. The ultrasonic loudspeaker is driven by a function generator, and the ultrasonic pressure applied to both sensors is 108 dB.

**Figure 7:**
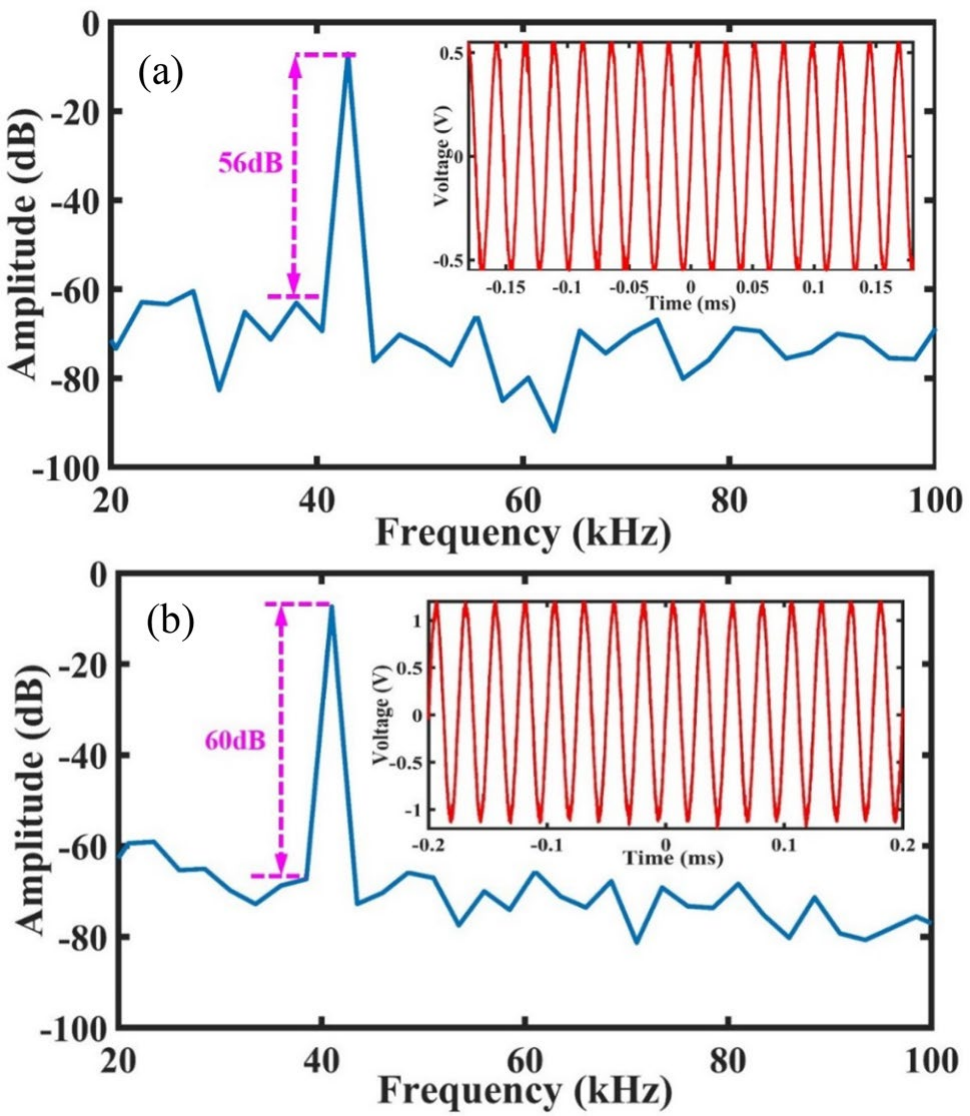
The time domain waveform and Fourier transformation of the EFPI sensor: (**a**) without FZP, and (**b**) with FZP.

**Figure 8:**
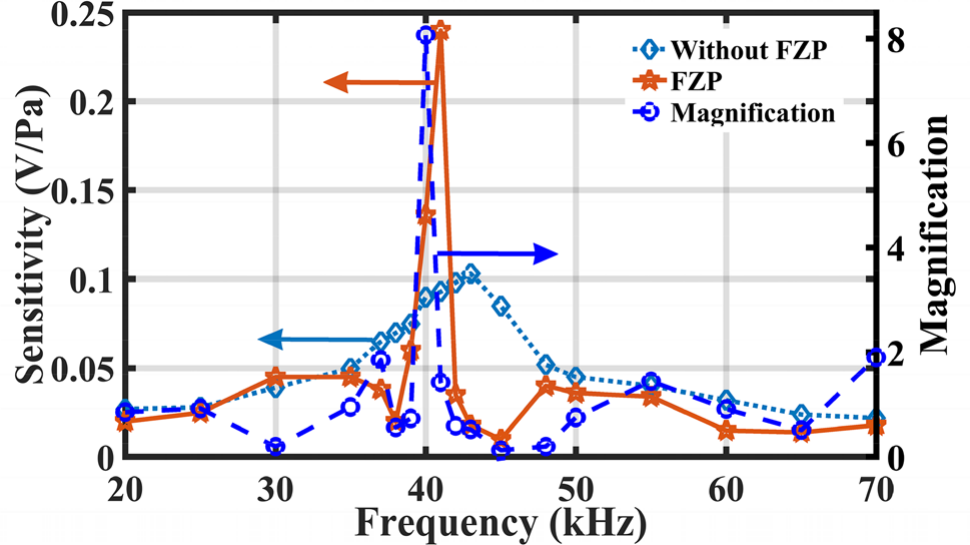
The sensitivity of the sensor with and without FZP versus ultrasonic frequency.

Considering the deviation caused by the differences in dimensions between the fabrication and design, the ultrasonic wave with frequencies ranging from 36 to 45 kHz is applied to the EFPI sensor to achieve the resonant frequency of beam-supported diaphragm and the focusing frequency of FZP, and the step is 1 kHz. The testing results show that when the EFPI sensor without FZP is excited by the 43-kHz-frequency ultrasonic wave, its responsive sensitivity is highest, and the values are − 19.8 dB re. 1 V/Pa. While the EFPI sensor with FZP obtains its maximum responsive sensitivity at the frequency of 41 kHz, and the corresponding value is − 12.4 dB re. 1 V/Pa. Figure 7a and b show the time domain waveforms and their Fourier transformation of the EFPI sensor with and without FZP at their resonant frequency, respectively. Similarly, the sensitivities of the sensor with and without FZP versus ultrasonic frequency are obtained. The results indicate that compared with the simulation results, the maximum magnification is shifted by 1 kHz, as is shown in Fig. 8, and the FZP achieves its focus functionality at the frequency of 41 kHz, thus causing a maximum responsive sensitivity to increase by 7.4 dB. Besides, the FZP leads to a narrower frequency band at the resonant frequency. In a word, the developed EFPI sensor with FZP shows a higher sensitivity and a narrower frequency band. In general, picking up the ultrasonic wave at a certain frequency is an important basis for judging whether there is partial discharge in a power system. As a result, the developed sensor has the great potential to enhance the capability for detecting the initially weak PDs.

## Conclusions

In summary, a novel EFPI sensor for PDs detection is demonstrated. The sensing diaphragm uses a beam-supported structure with the thickness of 5 μm, and is manufactured on a SOI wafer by using the MEMS processing technology, which is proven to be effective to reduce the beam-supported diaphragm manufacturing difficulty. Besides, the method of assembling a FZP inside the traditional probe structure to amplify the ultrasonic wave shows a good application prospect in enhancing the capability for detecting weak PDs, since it can not only increase the maximum responsive sensitivity, but also lead to a narrow bandwidth to eliminate the interference of ultrasonic waves to the detection signal at other frequencies. The final testing results show the maximum responsive sensitivity reaches to − 12.4 dB re. 1 V/Pa, which mainly benefits from the low thickness and damping ratio of beam-supported diaphragm and the application of FZP ultrasonic lens. In addition, this kind of sensing system also has great potentials for such applications as monitoring of specific failures, ultrasonic localization and other fields needing to detect ultrasonic waves at specific frequencies.
